# Empirical Analysis of Carbon Emission Accounting and Influencing Factors of Energy Consumption in China

**DOI:** 10.3390/ijerph15112467

**Published:** 2018-11-05

**Authors:** Zhaosu Meng, Huan Wang, Baona Wang

**Affiliations:** School of Economics, Ocean University of China, Qingdao 266100, China; wanghuan6730@stu.ouc.edu.cn (H.W.); wangbaona@stu.ouc.edu.cn (B.W.)

**Keywords:** fossil fuel, carbon emission, Logarithmic Mean Divisia Index (LMDI) method

## Abstract

China is confronting great pressure to reduce carbon emissions. This study focuses on the driving factors of carbon emissions in China using the Logarithmic Mean Divisia Index (LMDI) method. Seven economic factors, including gross domestic product (GDP), investment intensity, research and development (R&D) intensity, energy intensity, research and development (R&D) efficiency, energy structure and province structure are selected and the decomposition model of influencing factors of carbon emissions in China is constructed from a sectoral perspective. The influence of various economic factors on carbon emissions is analyzed quantitatively. Results show that the R&D intensity and energy intensity are the main factors inhibiting the growth of carbon emissions. GDP and investment intensity are the major factors promoting the growth of carbon emissions. The contribution of R&D efficiency to carbon emissions is decreasing. The impacts of energy structure and province structure on carbon emissions are ambiguous through time. Finally, some policy suggestions for strengthening the management of carbon emissions and carbon emission reduction are proposed.

## 1. Introduction

The Intergovernmental Panel on Climate Change (IPCC) Fifth Assessment Report (AR5) states that the evidence for global warming is unequivocal. The climate system including the atmosphere and oceans has warmed for more than one and a half century, leading to rising sea levels. These robust changes are caused by human activity, especially anthropogenic emissions of greenhouse gases (GHGs). This has caused global warming to become very serious in recent decades. It is well-known that the major GHG is carbon dioxide (CO_2_), which has contributed most of global warming since the Industrial Revolution [1]. The international community has been making great efforts to reduce carbon emissions to mitigate global warming [2,3].

China has been on an energy-intensive, heavy industry-based developmental pattern for decades, with high levels of GHGs being emitted. China has become the world’s largest carbon emitter since 2006 and has contributed 64.8% of the global carbon emission increments during 2007–2012 [4]. In 2013, 28% of all global carbon emissions were emitted from China, whose per capita emissions exceeded those of the European Union (EU) for the first time [5]. As a result, China plays a crucial role in tackling climate change.

The Chinese government is very active in exploring a practicable pathway of carbon emissions reduction, suitable for its industrial development and national conditions. In 2009, the Chinese government aimed for a 40–45% reduction in carbon emissions per unit of the gross domestic product (GDP) (carbon intensity) by 2020 relative to the 2005 level. A further commitment is to cut carbon intensity by 60–65% by 2030. However, according to the Chinese statistics year books, in 2016, China’s GDP was more than 74 trillion yuan with an increase of 6.7%. Industrial sectors, which create high pollution and high carbon emissions, contribute enormously to China’s economy. With industrialization and urbanization, the pressure of carbon emission reduction is increasingly huge. An analysis of the driving factors of carbon emissions and the contribution of each of these factors on CO_2_ emission intensity is crucial for both understanding the development of the CO_2_ emission problem and identifying appropriate approaches for mitigation of CO_2_ emissions in different regions of China.

There are plentiful studies focusing on the driving factors of China’s carbon emissions growth and the related strategies on a national scale [1,6,7,8,9,10,11,12,13] or from a provincial perspective [14,15,16,17,18]. At the factor decomposition level, a previous study [19] investigated carbon dioxide intensity in the power sector of twelve Asian countries and regions by using Divisia index decomposition approach methods and found that the structure and strength of the power sector are the main factors influencing CO_2_ emissions in China’s power sectors. Another study [20] used a factor decomposition method to suggest that CO_2_ emissions in the manufacturing sectors of China and Korea are affected by product structure, fuel share and sector energy intensity. China’s industrial CO_2_ emissions were also investigated based on four kinds of energy and eight industrial sectors [21].

**Figure 1:**
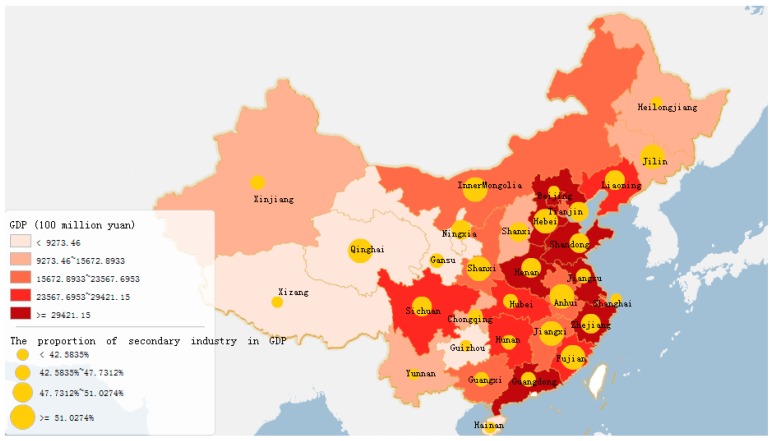
GDP growth rate and the proportion of secondary industry in GDP in the provincial level of China.

Moreover, as for the variation and dynamics of China’s economy in the provinces, municipalities and autonomous regions (collectively referred to as provinces), carbon emissions differ on the regional scale. To reach China’s emission reduction targets, the carbon emission growth in the various provinces needs to be effectively mitigated [22,23]. As illustrated in Figure 1, China’s provinces possess various socioeconomic development levels, industrial structures, energy consumption patterns and so on [23,24]. Some provinces in southeast China have entered the postindustrial stage, upgrading their industrial structures to be dominated by high-tech and tertiary industries. Meanwhile, some other provinces, especially in the middle China, northeast China and northwest China, are still in the process of industrialization, or rely on heavy industry [25,26,27]. In the case of energy consumption, fossil fuels are still the dominant source for most provinces in China, while in some provinces, local governments have made effort to gradually enhance the levels of clean energy utilization. Therefore, the growth trend of carbon emission and its driving factors reflect remarkable differentiation in a provincial scale [28].

To sum up, there are a number of studies focusing on the effects of GDP, energy intensity and energy structure on the increase of carbon emissions from the perspective of a certain industry (i.e., fishery and transportation) in the economy. Only few studies focus on the impact of research and development (R&D) on carbon emissions of China from a national perspective. With the ever-accelerating speed of technological development and technological innovation, R&D has become an important factor to promote economic development, and also an inevitable factor to consider in carbon emission reduction. This paper innovatively proposes variables such as R&D intensity and R&D efficiency and studies quantitatively their impacts on the growth of carbon emissions in China.

The objective of this study is to completely decompose China’s carbon emission increases into changes in related driving factors and explore the driving forces from a sectoral perspective. The carbon emission characteristics for different factors in China and the underlying drivers behind them are illustrated. Seven economic factors, including GDP, investment intensity, R&D intensity, energy intensity, R&D efficiency, energy structure and province structure are selected in this paper. The multi-layer Logarithmic Mean Divisia Index (LMDI) decomposition method is applied to investigate the driving factors which contributed to the growth in China’s carbon emissions between 2004 and 2014.

The paper is organized as follows: in the Introduction, we provide the background and significance of the study. A brief review of current studies on the driving forces of China’s carbon emissions are given. Next, we introduce the LMDI decomposition method and the datasets used. Then, the empirical results from the decomposition analysis on carbon emissions in China are presented, followed by suggestions on strengthening the management of carbon emission and carbon emission reduction are made to provide a reference for policymakers.

## 2. Methods and Data

### 2.1. The Logarithmic Mean Divisia Index (LMDI) Model

In the late 1970s, data decomposition methods started to be used. One important application is to analyze the relative contributions of predetermined factors to the change of an aggregate energy indicator. In many energy decomposition methods, there is a problem with unexplained residuals in the results [29]. The Index Decomposition Analysis method (IDA) is one of the energy research tools which has since been applied to other fields, such as CO_2_ emission analysis, environmental management, and sustainable use of natural resources. The basic idea of IDA is to decompose a target variable into a combination of different influencing factors, and then calculate the contribution of each influencing factor according to its calculation formula. The quantity and contribution rate are used to find out the relatively large factors affecting the target variable and distinguish the different effects of each factor on the target variable. Building upon IDA, many specific decomposition methods have arisen, and the Logarithmic Mean Divisia Index Method (LMDI) is one of the most commonly used. Since LMDI doesn’t have unexplained residual terms, but has time reversal and factor reversal properties, it is an appropriate method to analyze the driving factors of CO_2_ emissions in the different Chinese provinces [30]. Compared with other methods, LMDI performs particularly well not only when large variations exist in the values of variables, but also with zero values in a dataset [21,31] On a national, provincial or county level, the LMDI method is widely used for carbon emissions, changes in energy intensity and other fields [32,33]. Based on the previous literature, we chose the LMDI approach to decompose carbon emissions into seven factors from the 30 provinces in China.

### 2.2. Model Construction

The LMDI method has two forms, additive or multiplicative, respectively [34]. For this study, the LMDI additive decomposition method is applied. The upgrading of equipment and the progress of science and technology can have a great influence on energy consumption and thus have a certain impact on carbon emissions, so we use the extended LMDI equation in this paper to study the factors of the carbon emissions including nine major energy sources. The following formula is the equation for carbon emissions (Equation (1)):

(1) $$\begin{array}{ll} C & =\overset{30}{\underset{i=1}{\sum }}\overset{9}{\underset{j=1}{\sum }}G\times \frac{G_{i}}{G}\times \frac{I_{i}}{G_{i}}\times \frac{R_{i}}{I_{i}}\times \frac{G_{i}}{R_{i}}\times \frac{E_{i}}{G_{i}}\times \frac{E_{\mathrm{ij}}}{E_{i}}\times \frac{C_{\mathrm{ij}}\;}{E_{\mathrm{ij}}}\\ & =\overset{30}{\underset{i=1}{\sum }}\overset{9}{\underset{j=1}{\sum }}G\times {\mathrm{gg}}_{i}\times {\mathrm{ig}}_{i}\times {\mathrm{ri}}_{i}\times {\mathrm{gr}}_{i}\times {\mathrm{eg}}_{i}\times {\mathrm{ee}}_{\mathrm{ij}}\times {\mathrm{ce}}_{\mathrm{ij}} \end{array}$$

Table 1 summarizes the definitions of each variable used.

According to Equation (1), the factors to carbon emissions are decomposed into GDP, province structure, investment intensity, R&D intensity, R&D efficiency, energy intensity, energy structure and carbon emission factor. As stated by the IPCC assumption, the carbon emission coefficient of an energy source can be considered to be constant [35], so we can ignore this factor when studying changes in carbon emissions. The impact of the remaining seven factors on the changes of carbon emissions can be expressed by the following formula:

(2) ΔC=ΔG+Δgg+Δig+Δri+Δgr+Δeg+Δee

The effects of each factor on the increase in carbon emissions are as follows:

(3) $$\Delta G=\overset{30}{\underset{i=1}{\sum }}\overset{9}{\underset{j=1}{\sum }}L\left(C_{\mathrm{ij}}^{t-1},C_{\mathrm{ij}}^{t}\right)\ln\left[\frac{G_{\left(t\right)}}{G_{\left(t-1\right)}}\right]$$

(4) $$\Delta \mathrm{gg}=\overset{30}{\underset{i=1}{\sum }}\overset{9}{\underset{j=1}{\sum }}L\left(C_{\mathrm{ij}}^{t-1},C_{\mathrm{ij}}^{t}\right)\ln\left[\frac{{\mathrm{gg}}_{i\left(t\right)}}{{\mathrm{gg}}_{i\left(t-1\right)}}\right]$$

(5) $$\Delta \mathrm{ig}=\overset{30}{\underset{i=1}{\sum }}\overset{9}{\underset{j=1}{\sum }}L\left(C_{\mathrm{ij}}^{t-1},C_{\mathrm{ij}}^{t}\right)\ln\left[\frac{{\mathrm{ig}}_{i\left(t\right)}}{{\mathrm{ig}}_{i\left(t-1\right)}}\right]$$

(6) $$\Delta \mathrm{ri}=\overset{30}{\underset{i=1}{\sum }}\overset{9}{\underset{j=1}{\sum }}L\left(C_{\mathrm{ij}}^{t-1},C_{\mathrm{ij}}^{t}\right)\ln\left[\frac{{\mathrm{ri}}_{i\left(t\right)}}{{\mathrm{ri}}_{i\left(t-1\right)}}\right]$$

(7) $$\Delta \mathrm{gr}=\overset{30}{\underset{i=1}{\sum }}\overset{9}{\underset{j=1}{\sum }}L\left(C_{\mathrm{ij}}^{t-1},C_{\mathrm{ij}}^{t}\right)\ln\left[\frac{{\mathrm{gr}}_{i\left(t\right)}}{{\mathrm{gr}}_{i\left(t-1\right)}}\right]$$

(8) $$\Delta \mathrm{eg}=\overset{30}{\underset{i=1}{\sum }}\overset{9}{\underset{j=1}{\sum }}L\left(C_{\mathrm{ij}}^{t-1},C_{\mathrm{ij}}^{t}\right)\ln\left[\frac{{\mathrm{eg}}_{i\left(t\right)}}{{\mathrm{eg}}_{i\left(t-1\right)}}\right]$$

(9) $$\Delta \mathrm{ee}=\overset{30}{\underset{i=1}{\sum }}\overset{9}{\underset{j=1}{\sum }}L\left(C_{\mathrm{ij}}^{t-1},C_{\mathrm{ij}}^{t}\right)\ln\left[\frac{{\mathrm{ee}}_{\mathrm{ij}\left(t\right)}}{{\mathrm{ee}}_{\mathrm{ij}\left(t-1\right)}}\right]$$

In the above seven equations, $L\left(C_{\mathrm{ij}}^{t-1},C_{\mathrm{ij}}^{t}\right)$ is defined as follows:

$$L\left(C_{\mathrm{ij}}^{t-1},C_{\mathrm{ij}}^{t}\right)=\{\begin{array}{cc} \frac{C_{\mathrm{ij}}^{t}-C_{\mathrm{ij}}^{t-1}}{{\mathrm{lnC}}_{\mathrm{ij}}^{t}-{\mathrm{lnC}}_{\mathrm{ij}}^{t-1}}, & C_{\mathrm{ij}}^{t}\neq C_{\mathrm{ij}}^{t-1}\\ C_{\mathrm{ij}}^{t}\;\mathrm{or}\;C_{\mathrm{ij}}^{t-1}, & C_{\mathrm{ij}}^{t}=C_{\mathrm{ij}}^{t-1} \end{array}$$

In order to evaluate the total amount of carbon emissions, we calculate the carbon emission factors of different energy sources referring to the 2006 IPCC guidelines for national greenhouse gas inventories. Firstly, referring to the 2006 IPCC guidelines, the conversion rate whereby the calorific value of one kilogram of standard coal is about 29,271.2 kJ, we convert the energy consumption into a net calorific value. Secondly, the carbon content can be determined from the net calorific value for different energy categories. Thirdly, one trillion calorific value can be converted to 34.163 kg of standard coal. To get the carbon emission factors, we take the calculated carbon content and divide it by 34.163. The results are shown in Table 2.

The provincial carbon emission formula is as follows Equation (10):

(10) $$C=\overset{30}{\underset{i=1}{\sum }}\overset{9}{\underset{j=1}{\sum }}E_{\mathrm{ij}}\times \beta_{j}$$

where $E_{\mathrm{ij}}$ is the total consumption of energy j in province i. $\beta_{j}$ represents the carbon emission coefficient of energy j. Given the carbon emission coefficient of an energy source is constant, which means that the carbon emission coefficient doesn’t vary for different provinces, $\beta_{j}$ is approximately equal to ${\mathrm{ce}}_{\mathrm{ij}}$ in this equation. C is the total carbon emissions of the chosen 30 provinces.

### 2.3. Data

Provincial level panel data is used for the sectoral perspective. Considering the data availability, we focus on 22 provinces, four municipalities and four autonomous regions from 2004 to 2014 (Table 3). Four provinces, Hong Kong, Macao, Taiwan and Tibet, are excluded from the analysis due to data unavailability.

In this paper, the data for GDP and the fixed asset investments are collected from the China Statistical Yearbooks (2005–2015). The R&D expenditure data are derived from China Science and Technology Statistics Yearbooks (2005–2015). According to the China Energy Statistics Yearbooks (2005–2015), we divide energy into nine categories, including raw coal, coke, crude oil, gasoline, kerosene, diesel, fuel oil, liquefied petroleum gas (LPG) and natural gas (Table 4). All energy consumptions are converted into standard coal consumption. We replace all zero values in the data set with 10–100.

## 3. Empirical Results

### 3.1. Carbon Emissions and Energy Consumption Structure

**Figure 2:**
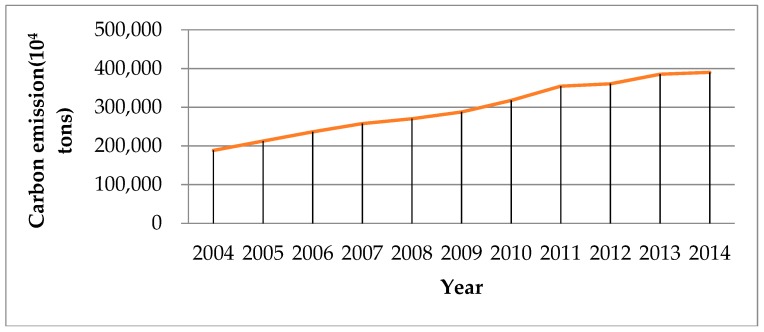
The total amount of carbon emissions from 2004 to 2014.

**Figure 3:**
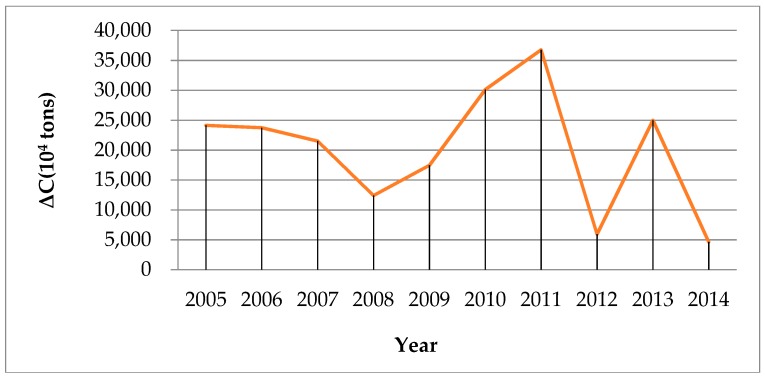
The increment of carbon emissions (ΔC) from 2005 to 2014.

In the last decade, China has been undergoing a distinct and exceptional phase. The growth of energy-intensive heavy industries led to a large amount of greenhouse gases, which brought huge negative impacts to the environment. From 2004 to 2014, carbon emissions in China increased by 107%. The absolute increment reached 2016.38 Mt CO_2_. The total amount of carbon emissions (Figure 2) is greatly affected by economic activity, energy intensity and energy structure changes. The increment of carbon emissions from 2005 to 2014 is highly volatile (Figure 3). The 2008–2012 global financial crisis had a great influence on the economy, particularly in the manufacturing sector. Therefore, enterprises tended to reduce the scale of production which slowed down the increment of carbon emissions, and there is a significant drop in the carbon emission increase in 2012 due to the international financial crisis. Statistics show that in 2010 and 2011, the bilateral trade volume between the EU and China has increased by 31.8% and 18.3%, respectively, compared with last year. However, from January to May 2012, the bilateral trade volume between the EU and China increased by only 1.3%, compared with the previous year. It can be seen that the international financial crisis had a great negative impact on China’s imports and exports. The decline in exports led to a reduction of production in China and the increment of carbon emissions was correspondingly smaller. Meanwhile, in order to achieve its emission reduction targets, the Chinese government has adopted a series of policies and gradually updated the industrial structure, focusing on low carbon emissions. In 2011 and 2012, the Chinese government issued a number of new energy conservation, emission reduction and environmental protection policies, including the “12th Five-Year plan” on Energy Saving and Emission Reduction. The volatility of the increment of carbon emissions during 2011–2013 is very likely affected by the international financial crisis, but we can find that in the long run, there is a downward trend in the increment of carbon emissions since 2011 which suggests a long-lasting effect of government policies. However, it is still noticeable that the annual total carbon emissions are still at a high level, which reached 390,101.98 × 10^4^ tons.

**Figure 4:**
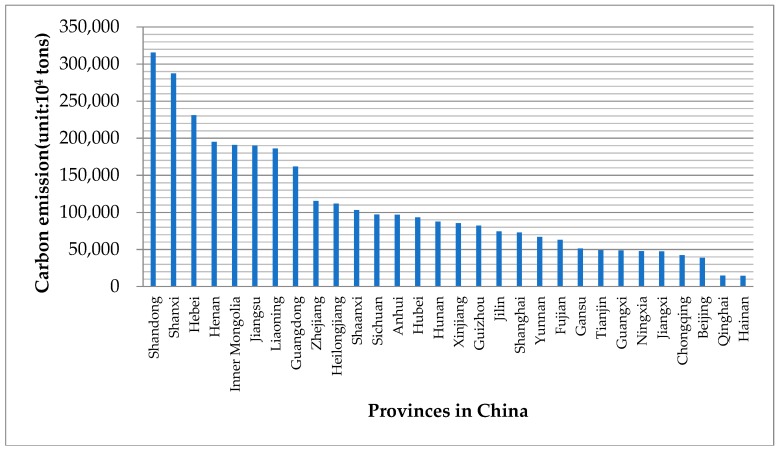
Accumulative carbon emissions from 2004 to 2014 for 30 provinces (districts) in China.

The cumulative carbon emissions from 2004 to 2014 for the 30 districts are illustrated in Figure 4. The contribution of provinces (or districts) to the increased national carbon emissions varied between 2004 and 2014 due to the differences in socioeconomic development levels, industrial structure, energy consumption patterns and so on. Shandong is the top carbon emission province, reaching 3154.91 million tons. Secondary industry activities represented 48.44% of Shandong’s GDP in 2014. The industrial orientation is to neighboring-port services, shipbuilding, heavy industries and so on. Next to Shandong are Shanxi, Hebei, Henan, Inner Mongolia and Jiangsu. The top six provinces contributed 43.22% of China’s total carbon emissions either for an ongoing industrialization process or for being heavily reliant on heavy industry. Liaoning, Guangdong, Zhejiang and Heilongjiang are the next four provincial drivers in descending order. On the contrary, the carbon emissions of the remaining 20 provinces are below the national average, i.e., Beijing and Hainan. These provinces are either focused on high-tech and in a postindustrial stage (Beijing) or mainly rely on tertiary industry (Hainan). To sum up, according to the economic development level and industrial structure evolution, significant differences can be found among the individual contributions of the 30 studied districts to the growth of China’s national carbon emissions.

The energy consumption structure in 2004–2014 is shown in Table 5. The overall energy consumption structure was stable. Coal ranked first among all the energy consumption, accounting for more than 60%. The next is crude oil, which accounted for nearly 14% but with a decreasing tendency. The remaining seven kinds of energy accounted for about 20%. In order to reduce carbon emissions and achieve environment-friendly development, the government encourages the use of low-carbon energy sources and energy saving resolutions.

### 3.2. Decomposition of Carbon Emission Factors

Carbon emissions are greatly increased due to the effect of economic expansion, while energy structure plays an important role in abating them, so we use the extended LMDI equation in this paper to study the factors of the carbon emissions from nine major energy sources. Using the extended LMDI model, we are able to decompose the carbon emissions from 30 provinces in China into seven factors. Table 6 and Table 7 show the year-by-year effects and the cumulative effects of GDP, province structure, investment intensity, R&D intensity, R&D efficiency, energy intensity and energy structure.

From the year-to-year analysis, we find that GDP and energy intensity have a huge and consistent impact on carbon emission through time, while GDP has a large and profound positive effect, and energy intensity has a negative effect. Investment intensity has a positive effect except for 2011. R&D intensity has a negative effect except for 2011. The effect of energy structure is ambiguous. Table 7 shows that GDP, province structure, investment intensity and energy structure all have positive impact on the change of carbon emissions, while R&D intensity and energy intensity have negative impact on the change of carbon emissions. R&D efficiency has a positive effect after 2009.

**Figure 5:**
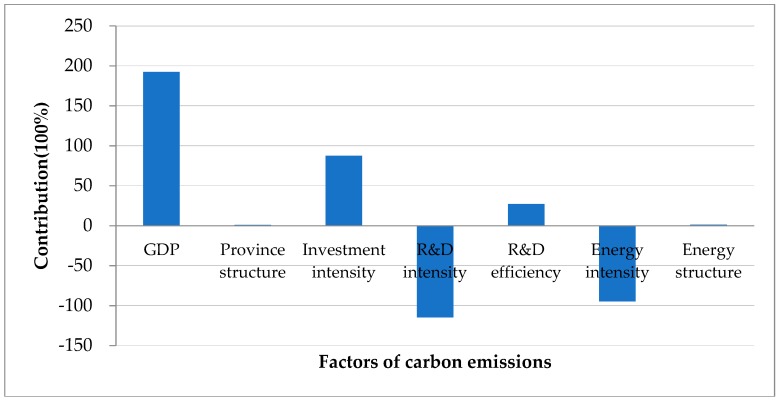
Accumulated contributions of different factors of carbon emissions on energy consumption of China in 2014.

**Figure 6:**
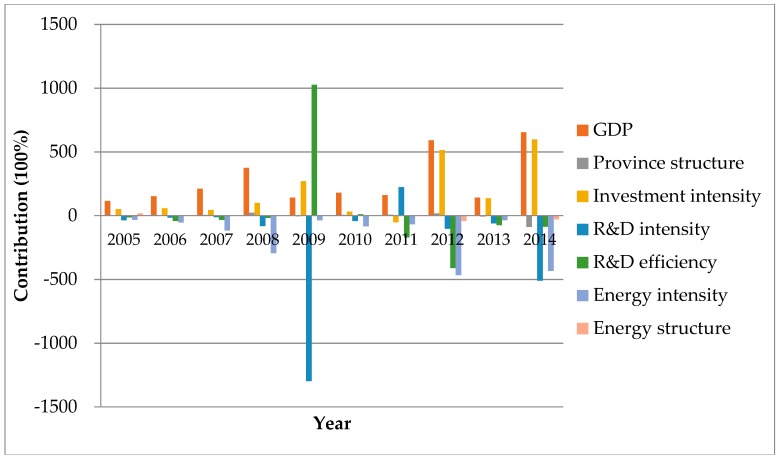
The year-by-year contributions of different factors of carbon emissions from energy consumption of China in 2005–2014.

The cumulative contributions and the annual contributions of GDP, province structure and the other five factors are given in Figure 5 and Figure 6. Using the contribution percentage for quantification, the contributions of GDP, province structure, investment intensity, R&D intensity, R&D efficiency, energy intensity and energy structure are 192.54%, 0.94%, 87.35%, −114.59%, 27.24%, −94.77% and 1.29%, respectively (Figure 5). From the absolute value of the contributions, we can find that the change of carbon emissions is mainly due to GDP, investment intensity, R&D intensity and energy intensity. Relatively speaking, the absolute value of contributions for province structure, R&D efficiency and energy structure are small, which means they only have small impacts on the changes of carbon emissions.

The annual contributions of seven factors to carbon emissions are illustrated in Figure 6. The impact of GDP is always positive and at a high level, which means that GDP is the main positive driving factor of the carbon emissions. Beside GDP, the influence of investment intensity is obvious and, on a rise, especially in the period between 2012 and 2014. As investment increases, factories are tending to expand production and increase energy consumption, which eventually results to large carbon emissions. Referring to Table 6, it’s explicit that the main negative drivers of carbon emissions are R&D intensity and energy intensity. R&D activities promote technological progress, while energy intensity effect captures the effectiveness of investments for energy savings, the improvement of technology, and efficiency of policies. Both of them can be viewed as offsets of the carbon emissions.

**Figure 7:**
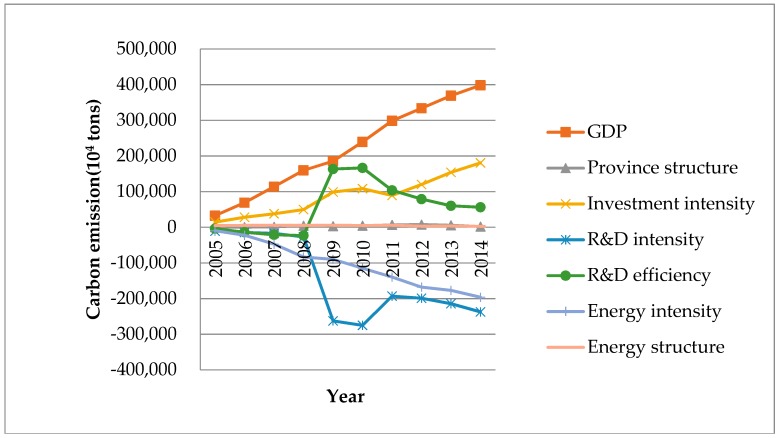
The accumulated effect of different factors of carbon emissions from energy consumption of China in 2005–2014.

Figure 7 illustrates the accumulated effect of each factor on the changes in carbon emissions. GDP and energy structure all play a positive role in the change of carbon emissions. The province structure shows a negative impact in year 2006, 2009, 2013 and 2014. In other years it shows a positive impact. In summary, the cumulative result is positive. In this period, except for 2011, the investment intensity shows a positive impact on the change of carbon emissions, and the cumulative result is positive. R&D intensity and energy intensity show negative impacts on the change. Between 2009 and 2010, the R&D efficiency plays a positive role, but in the other eight years, it converts to a negative impact. The cumulative value is positive.

The province structure has a positive cumulative impact on the change of carbon emissions, but its impact is not stable. In 2013 and 2014, the impacts are negative, which means that the change of regional economic structure has played a role in curbing the growth of carbon emissions, suggesting we can control carbon emission increases by optimizing the regional economic structures.

**Figure 8:**
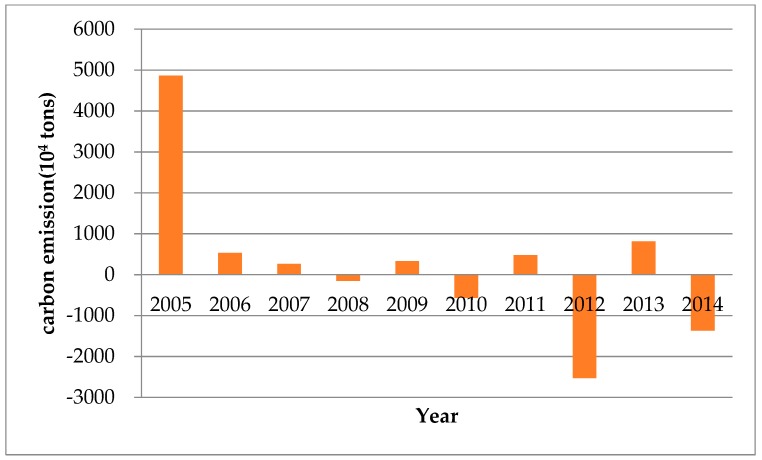
The effect of energy structure on carbon emissions in China during 2005–2014.

The effect of energy structure volatiles through time. In 2005–2007, 2009, 2011 and 2013, the contributions are positive, the rest of the year are negative (Figure 8). This trend is basically in line with the changing trend of high carbon energy, such as raw coal, clean coal, coal products and coke. As far as contribution is concerned, the absolute value of the energy consumption structure contributes year by year is relatively low, indicating that China can optimize the energy consumption structure and improve the offsetting ability of carbon emissions from rapid economic growth.

## 4. Discussion and Conclusions

### 4.1. Discussion

We decompose the carbon emissions from 30 districts in China into seven factors. The factors in the decomposition of carbon emissions are GDP, investment intensity, R&D intensity, energy intensity, R&D efficiency, energy structure, and province structure. In terms of the accumulated effects of the seven factors, the growth of GDP is the biggest driver of the carbon emission increment, which accounts for 192.54%, which indicates that China’s economic development mainly depends on energy consumption. At present, China is in a period of major structural transformation and is moving towards a new model of balanced and coordinated development. How to keep carbon emissions under control while maintaining a satisfactory economic growth has become a current issue that must be solved.

Investment intensity also plays an important role in carbon emission increments. As investment increases, expending reproduction happens and energy consumption increases, resulting in more carbon emissions. In our opinion, the crux of the matter lies not in the increment of investment, but rather the direction of the increased investment. If money is invested in heavily polluting industries with low level of production technologies and heavy energy consumption, the increase in investment will unavoidably lead to a carbon emission increment. However, if money is invested in industries that use clean energy or upgrading industrial structures in high-energy-intensity industries, this increase in investment intensity can actually help reduce carbon emissions.

Among the carbon emission factors, the influence of R&D intensity and energy intensity is significant. According to the year-by-year decomposition results, both factors have big contribution to carbon emission reductions which means they can be viewed as offsetting the increase energy consumption from economic growth. The energy intensity is a very powerful factor to curb carbon emissions as China has always attached great importance to energy conservation and emission reduction. The technical efficiency and resource utilization efficiency have been greatly improved, which makes the energy intensity, especially the energy intensity of the secondary industry, continue to decline. Besides, China has undertaken wide-ranging efforts to increase R&D investment in industries and has adopted energy efficiency standards to help reduce carbon emissions.

The impact of R&D efficiency on carbon emissions is not consistent through time. The outcomes of R&D are not immediately apparent. Additionally, firms need a long-term perspective on innovation, as R&D cannot always completely offset the cost of compliance. Generally, the debate on the link between R&D efficiency and carbon emission continues and lacks consensus. Our results reveal that the contribution of R&D efficiency to carbon emissions is decreasing, especially after 2009, which proves that government encouragement and guidance to corporate managers on how to achieve superior environmental performance and economic performance simultaneously is working.

Energy structure and province structure have no obvious inhibition effect on carbon emissions, which is similar to the results of some other studies [35]. The changes in the energy structure have been somewhat reduced carbon emissions in the industrial sector in certain years. From 2005 to 2014, except for Beijing, Shanghai and Hainan, the proportion of the secondary industry in most areas of China has always been between 45% and 60%, and coal is the main energy source. This shows that the economic growth of various regions still depends strongly on the secondary industry, so it is still a heavy task to adjust the industrial structure in the future. As the regional differences are obvious, there is still a long way to go to adjust the industrial structure and energy structure.

### 4.2. Conclusions and Future Perspectives

In the last decade, China was undergoing a distinct and exceptional phase, during which high levels of greenhouse gases emitted were linked closely with the energy-intensive, heavy industry-based economic growth pattern. However, the 13th Five-Year-Plan of China for economic development (2016–2020) is finalized, which set new goals on achieving more sustainable and inclusive growth. This study analyzes carbon emissions and the influencing factors of energy consumption in China using the LMDI method. We innovatively introduce R&D intensity and R&D efficiency into the empirical study, and main findings are as follows:

Carbon emissions in China increased dramatically between 2004 and 2014. The effect of economic expansion (GDP growth and investment intensity) dominantly drives up carbon emissions, accounting for about a 279.89% increase. R&D intensity and energy intensity have the most significant influence on carbon emission reductions, accounting for −114.59% and −94.77% respectively. The contribution of R&D efficiency to carbon emission is decreasing, especially after 2009 which reveals that the government’s guidance on green and sustainable business is working. The impacts of energy structure and province structure on carbon emissions are ambiguous.

These major findings indicate a rapid rise in carbon emissions combined with fast GDP growth from 2005 to 2014. During the acceleration in heavy industrialization, a great amount of investment went into heavy industries, especially the traditional polluting industries. China’s rapid development has sacrificed too much energy, leading to huge amounts of CO_2_ emissions. Against the background of reducing global carbon emission and improving the atmospheric environment, this paper helps analyze the driving factors of carbon emission of different factors as well as the emissions in different allocations. According to the discussion above, we suggest the following policy options for designing low-carbon development strategies for related provinces of China:

Adjusting the energy consumption structure is crucial for energy saving and carbon emissions reduction. To maintain and expand its offset effect on carbon emissions, we need to encourage energy adaptation, increase R&D investment with sustainable and environmentally friendly features, improve the energy structure, promote the marketization of the energy system and the replacement of the traditional fossil energy sources with new energy forms. Inspections, supervision and law enforcement of relevant departments should be conducted, and multiple economic instruments should be applied. In decision-making process, environment bearing capacity should be considered to improve the efficiency and effectiveness of the policy for carbon emission reduction.

This paper is subject to some limitations. Instead of dividing R&D into general R&D and green R&D, the study takes R&D intensity as a whole, due to data availability. Besides, for different industries, the green R&D activities vary and may need deeper investigation. Second, the study covers the period of 2004–2014 in China. Since national environmental regulations and policies as well as market pressures can influence R&D and energy intensities, we should be cautious when comparing with other countries. Future investigation could be directed at broadening the model to further consider important variables such as national environmental regulations.

## Figures and Tables

**Table 1:** Variable indices.

Variable	Definition	Variable	Definition
i	Area	j	Fuel
G	Gross domestic product	ggi	Province structure: GDP share of area i
Gi	Gross domestic product of area i	igi	Investment intensity: share of fixed asset investment in GDP of area i
Ii	Fixed asset investment of area i	rii	R&D intensity: share of R&D Expenditure in fixed asset investment of area i
Ri	R&D expenditure of area i	gri	R&D efficiency: GDP per unit of R&D expenditure in area i
Ei	Gross energy consumption of area i	egi	Energy intensity: gross energy consumption per unit of GDP in area i
Eij	Consumption of fuel j in area i	eeij	Energy structure: share of Consumption of fuel j in gross energy consumption in area i
Cij	Carbon emission by fuel j in area i	ceij	Carbon emission coefficient: carbon emission per unit of fuel j in area i

**Table 2:** Carbon emission factors of different energy sources.

Energy (104 tons)	Coal	Coke	Crude Oil	Gasoline	Kerosene	Diesel Oil	Fuel Oil	LPG	Natural Gas
Standard coal coefficient	0.7143	0.9714	1.4286	1.4714	1.4714	1.4571	1.4286	1.7143	1.3300
Net calorific value (TJ/Gg)	20.9084	28.4340	41.8168	43.0696	43.0696	42.6511	41.8168	50.1796	38.9307
Carbon content (kg/GJ)	26.2000	29.2000	20.0000	19.0000	19.6000	20.2000	21.1000	17.2000	15.3000
Carbon emission factor	0.7668	0.8546	0.5854	0.5561	0.5737	0.5912	0.6176	0.5034	0.4478

**Table 3:** Indices for 30 districts.

Index	Province	Index	Province
1	Henan	16	Jiangxi
2	Hebei	17	Shaanxi
3	Shanxi	18	Hainan
4	Shandong	19	Jiangsu
5	Hunan	20	Anhui
6	Hubei	21	Yunnan
7	Sichuan	22	Guizhou
8	Qinghai	23	Beijing
9	Jilin	24	Tianjin
10	Liaoning	25	Shanghai
11	Heilongjiang	26	Chongqing
12	Guangdong	27	Xinjiang
13	Zhejiang	28	Inner Mongolia
14	Fujian	29	Guangxi
15	Gansu	30	Ningxia

**Table 4:** Indices for the energy subcategories.

Index	Category	Index	Category
1	raw coal	6	diesel
2	coke	7	fuel oil
3	crude oil	8	liquefied petroleum gas
4	gasoline	9	natural gas
5	kerosene		

**Table 5:** The structure of energy consumption of China in 2004–2014.

Year	Coal	Coke	Crude Oil	Gasoline	Kerosene	Diesel Oil	Fuel Oil	LAG	Natural Gas
2004	64.31%	6.71%	15.61%	2.77%	0.53%	4.58%	2.26%	1.18%	2.06%
2005	63.51%	7.38%	14.88%	3.02%	0.54%	5.24%	2.10%	1.26%	2.07%
2006	63.43%	7.93%	14.30%	3.03%	0.57%	5.30%	1.85%	1.15%	2.44%
2007	63.80%	7.89%	14.00%	3.11%	0.57%	5.25%	1.60%	1.12%	2.64%
2008	63.73%	7.97%	13.74%	3.06%	0.60%	5.41%	1.47%	1.10%	2.92%
2009	63.37%	8.05%	13.92%	3.07%	0.60%	5.40%	1.32%	1.06%	3.21%
2010	62.82%	7.92%	14.17%	3.18%	0.61%	5.37%	1.46%	1.01%	3.46%
2011	64.04%	7.75%	13.36%	3.19%	0.59%	5.18%	1.35%	1.01%	3.55%
2012	62.97%	7.92%	13.51%	3.38%	0.64%	5.32%	1.33%	0.98%	3.96%
2013	64.85%	7.34%	12.97%	3.12%	0.64%	4.77%	1.33%	0.90%	4.07%
2014	63.81%	7.35%	13.42%	3.21%	0.69%	4.76%	1.39%	0.93%	4.44%

**Table 6:** The year-by-year effects of different factors of carbon emissions from energy consumption of China in 2005–2014 (unit: 10^4^ tons).

Year	GDP	Province Structure	Investment Intensity	R&D Intensity	R&D Efficiency	Energy Intensity	Energy Structure	Total
2005	32733.32	578.73	14612.34	−10716.93	−3895.41	−9495.71	4861.29	28677.64
2006	36248.18	−59.97	13719.83	−3686.84	−10032.99	−12806.90	534.29	23915.60
2007	44940.65	922.60	9438.10	−2496.45	−6941.65	−24749.78	260.87	21374.34
2008	45915.20	2694.02	12113.59	−9878.65	−2234.94	−36176.33	−149.51	12283.39
2009	25602.51	−1062.48	49088.18	−235736.01	186647.84	−6701.53	332.51	18171.00
2010	53977.13	1360.51	9346.25	−12443.74	3097.50	−24701.53	−568.29	30067.82
2011	59153.88	2341.72	−19064.64	82076.78	−63012.14	−25076.07	478.15	36897.69
2012	35446.10	1020.09	30784.08	−6164.43	−24619.65	−27938.93	−2529.92	5997.34
2013	35123.89	−1864.67	33956.55	−15185.74	−18770.81	−9021.53	815.45	25053.13
2014	29264.15	−3989.50	26750.61	−22881.40	−3869.21	−19426.68	−1366.38	4481.60

**Table 7:** The accumulated effects of different factors of carbon emissions from energy consumption of China in 2005–2014 (unit: 10^4^ tons).

Year	GDP	Province Structure	Investment Intensity	R&D Intensity	R&D Efficiency	Energy Intensity	Energy Structure	Total
2005	32733.32	578.73	14612.34	−10716.93	−3895.41	−9495.71	4861.29	28677.64
2006	68981.50	518.77	28332.17	−14403.77	−13928.40	−22302.61	5395.58	52593.24
2007	113922.15	1441.37	37770.27	−16900.23	-20870.04	−47052.38	5656.44	73967.58
2008	159837.35	4135.39	49883.86	−26778.87	-23104.99	−83228.71	5506.93	86250.97
2009	185439.86	3072.91	98972.03	−262514.88	163542.85	-89930.25	5839.45	104421.97
2010	239416.99	4433.42	108318.28	−274958.63	166640.35	−114631.77	5271.16	134489.79
2011	298570.87	6775.14	89253.64	−192881.85	103628.21	−139707.84	5749.31	171387.48
2012	334016.97	7795.23	120037.72	−199046.28	79008.56	−167646.77	3219.39	177384.82
2013	369140.86	5930.55	153994.27	−214232.01	60237.75	−176668.30	4034.84	202437.95
2014	398405.01	1941.05	180744.88	−237113.41	56368.53	−196094.98	2668.46	206919.55
